# The Role of Vasoactive Intestinal Peptide in Glucagon-like Peptide-2-Mediated Intestinal Lipid Handling

**DOI:** 10.3390/ijms27125457

**Published:** 2026-06-17

**Authors:** Kundanika Mukherjee, Rita Wang, Farnoosh Tabatabaeian, Changting Xiao

**Affiliations:** Department of Anatomy, Physiology and Pharmacology, College of Medicine, University of Saskatchewan, 107 Wiggins Road, Saskatoon, SK S7N 5E5, Canada; kum581@mail.usask.ca (K.M.); rita.wang@usask.ca (R.W.); farnoosh.tab@usask.ca (F.T.)

**Keywords:** vasoactive intestinal peptide, glucagon-like peptide-2, chylomicrons, IL-22

## Abstract

The gut hormone glucagon-like peptide-2 (GLP-2) plays important roles in regulating lipid handling and promoting anti-inflammatory functions in the intestine. During the postprandial state, it increases lipid absorption. During post-absorptive state, it mobilizes pre-formed chylomicrons. GLP-2 acts through vasoactive intestinal peptide (VIP) in reducing inflammation in rat ileum. However, this pathway has not yet been tested for GLP-2’s effects on intestinal lipid handling. Here, in mesenteric lymph duct cannulated rats, we examined whether VIP signaling mediates GLP-2’s effects on postprandial lipid absorption and post-absorptive lipid mobilization in the intestine. We administered a VIP receptor antagonist and analyzed lipid output in response to intraperitoneal GLP-2 or PBS during postprandial and post-absorptive states. VIP receptor antagonism reduced GLP-2 mediated lipid output in the post-absorptive state but had no effect during the postprandial state. These results show that GLP-2 functions differently during postprandial and post-absorptive states and VIP aids in GLP-2-mediated lipid output during the post-absorptive state.

## 1. Introduction

Nutrient ingestion leads to the secretion of the gut hormone glucagon-like peptide-2 (GLP-2), a product of the proglucagon gene. Studies have shown that GLP-2 plays important roles in intestinal lipid handling during both the postprandial state and the post-absorptive state but with notable differences. During the postprandial state, GLP-2 increases lipid absorption and chylomicron formation in intestinal absorptive cells (enterocytes) [1,2]. During the post-absorptive state, GLP-2 releases “pre-formed” chylomicrons stored in intestinal locations outside the enterocytes [3,4,5].

The mechanism whereby GLP-2 regulates intestinal lipid handling is complex (reviewed in [6,7]). Several lines of evidence point to the involvement of the enteric nervous system (ENS). GLP-2 receptor (GLP-2R) is expressed on enteric neurons [8]. The ENS has been shown to mediate the regulation of intestinal lipid handling by GLP-2. GLP-2 was shown to stimulate postprandial lipid absorption via neuronal nitric oxide synthase (NOS), where neuronal NOS knockout in mice or neuronal NOS inhibition in hamsters abolished the effects of exogenous GLP-2 on lipid absorption and chylomicron production [9]. GLP-2 acted through the ENS in mobilizing gut lipids, as GLP-2’s effect on enhancing lipid output in fasted rats was abolished with an ENS inhibitor [10]. Despite these advances, the ENS components that mediate GLP-2 regulation of lipid handling in the intestine remain poorly defined.

A potential link emerges between GLP-2’s actions on lipid handling and vasoactive intestinal peptide (VIP). VIP is a 28-amino acid neuropeptide belonging to the glucagon-secretin superfamily of peptides [11]. GLP-2 stimulates VIP secretion from enteric VIP-expressing neurons [12]. Enterocytes are known to express VIP receptors (VIPRs) [13,14,15]. GLP-2’s anti-inflammatory effects involve the activation of VIP neurons in the submucosal ganglia of rat ileum [16]. In a recent study in mice, it was shown that intestinal luminal lipids stimulate VIP-expressing neurons to release VIP, which activates VIPRs on type-3 innate lymphoid cells to inhibit interleukin-22 (IL-22) production and subsequently enhance lipid absorption [17]. This increase in lipid absorption was accompanied by upregulation of lipid-binding proteins and transporters including Fabp2 and Cd36 and reduction in antimicrobial activity. Since nutrient ingestion stimulates GLP-2 secretion, it is likely that endogenous GLP-2 stimulates VIP secretion to enhance lipid absorption during the postprandial state. During fasted state where luminal nutrients are absent, exogenous GLP-2 might stimulate VIP secretion to release intestinally stored lipids, which has not been tested. It remains unknown whether VIP signaling responds to GLP-2 stimulation to enhance lipid secretion from the intestine. Here in this study in mesenteric lymph duct cannulated rats and with pharmacological targeting of VIPRs, we examined the involvement of VIP signaling in the regulation of intestinal lipid handling by GLP-2, especially on chylomicron release during fasted state.

## 2. Results

### 2.1. Exogenous VIP Had Minimal Effects on Intestinal Lymph Flow Rate, Chylomicron Output, and IL-22 Secretion During Postprandial State

**Figure 1:**
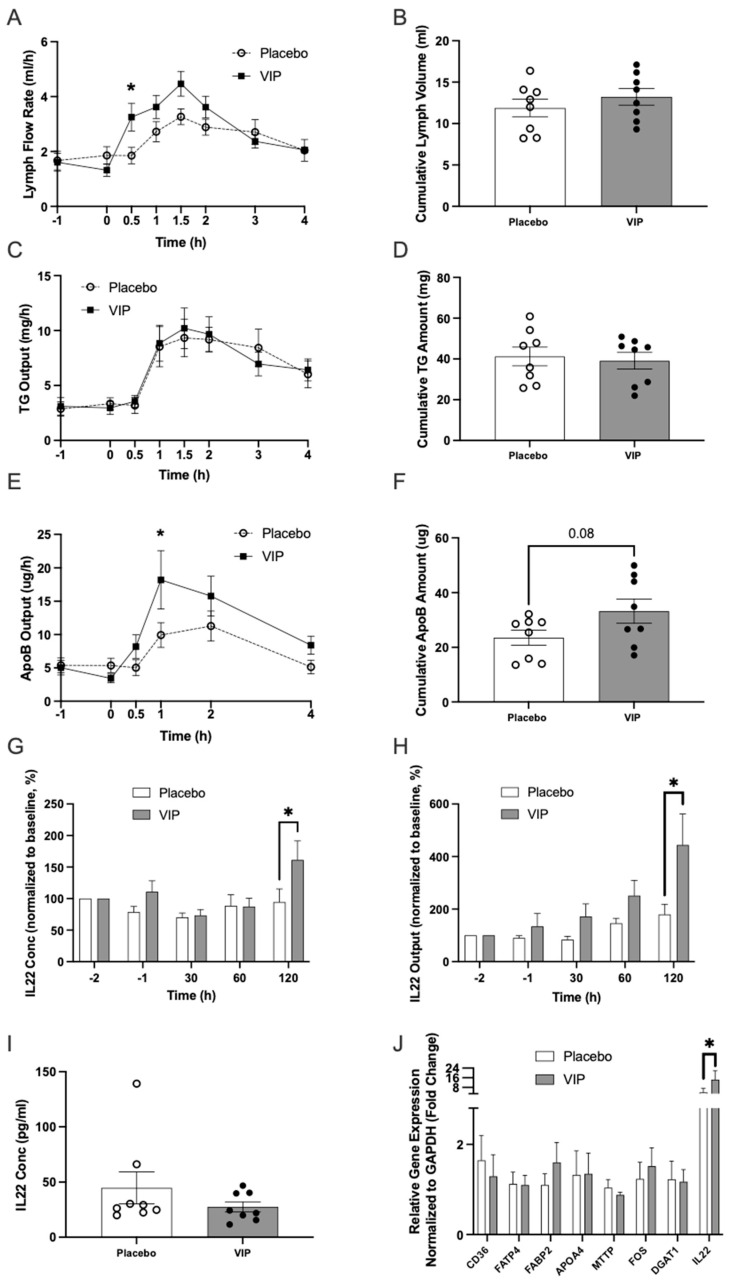
Effects of exogenous VIP treatment on lipid absorption, IL−22 secretion, and gene expression in the intestine in rats. (**A**) LFR over time following i.p. VIP or placebo treatment in rats. LFR was measured by collecting lymph for 4 h. (**B**) Cumulative lymph volume following i.p. VIP or placebo treatment in rats. (**C**) TG output and (**D**) cumulative TG amount in lymph were measured over 4 h. (**E**) ApoB output and (**F**) cumulative ApoB amount in lymph over time following i.p. VIP or placebo treatment. (**G**) IL−22 concentration and (**H**) IL−22 output in lymph over time following i.p. VIP or placebo treatment, expressed as % changed from baseline (−2 h). (**I**) IL−22 concentration in plasma following i.p. VIP or placebo treatment. (**J**) mRNA expression patterns of *Cd36*, *Fatp4*, *Fabp2*, *Apoa4*, *Mttp*, *Fos*, *Dgat1* and *Il22* following i.p. VIP or placebo treatment. Results are expressed as mean ± SEM: i.p. VIP, n = 8; i.p. PBS, n = 8. * *p* < 0.05 VIP vs. placebo. For clarity, statistical significance for increases from baseline within each treatment is not shown.

The first experiment examined whether exogenous VIP affects lipid absorption during fed state. Lymph flow rate (LFR) reflects intestinal secretion of lymph fluid which contains lipids and lipoproteins. LFR in rats treated with VIP was significantly higher than in PBS-treated rats at 30 min (Figure 1A). Cumulative lymph volume (equivalent to area-under-the-curve) was not different between groups (Figure 1B). Triglyceride (TG) output and cumulative TG amount were not significantly different between groups (Figure 1C,D). Apolipoprotein B (ApoB) output was significantly higher in VIP-treated groups compared to the control group (placebo) at 1 h post-treatment (Figure 1E). Cumulative apoB amount was not significantly different between groups (Figure 1F). IL-22 concentration and output in lymph were significantly higher in the VIP group compared to the placebo group at 120 min following treatment (Figure 1G,H). IL-22 concentrations in plasma were not significantly different between groups (Figure 1I). Il22 mRNA expression was significantly higher in the VIP-treated group compared to the placebo group (Figure 1J).

### 2.2. Antagonizing VIP Receptors Did Not Affect GLP-2-Mediated Lymph Flow, Lipid Output or IL-22 Secretion During Postprandial State

**Figure 2:**
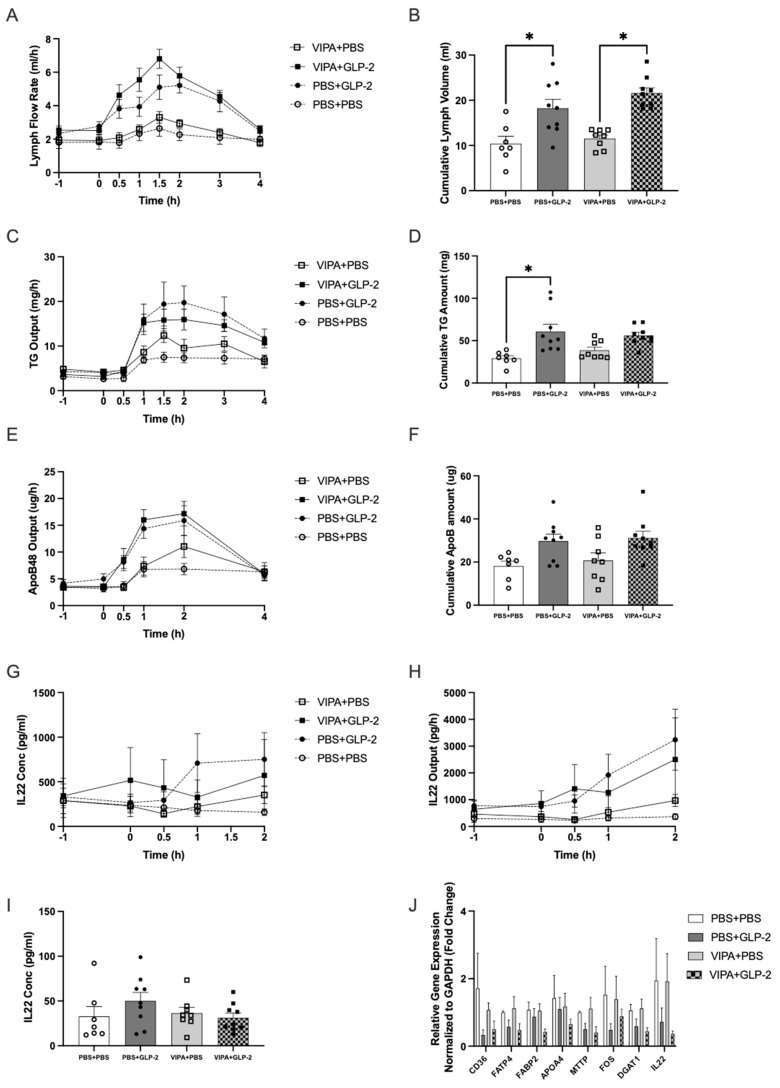
Effect of exogenous GLP−2 treatment in rats with VIP receptor antagonism on lipid absorption, IL−22 secretion, and gene expression in the intestine in rats. (**A**) LFR over time. LFR was measured by collecting lymph for 4 h. (**B**) Cumulative lymph volume. (**C**) TG output and (**D**) cumulative TG amount in lymph were measured over 4 h. (**E**) ApoB output and (**F**) cumulative ApoB amount in lymph over time. (**G**) IL−22 concentration and (**H**) IL−22 output in lymph over time. (**I**) IL−22 concentration in plasma. Plasma was collected at the end of experiment, t = 4 h. (**J**) mRNA expression patterns of *Cd36*, *Fatp4*, *Fabp2*, *Apoa4*, *Mttp*, *Fos*, *Dgat1* and *Il22*. Results are expressed as mean ± SEM: VIPA+PBS, n = 8; VIPA+GLP−2, n = 9; PBS+GLP−2, n = 9; PBS+PBS, n = 7. * *p* < 0.05. For clarity, statistical significance for increases from baseline within each treatment is not shown.

The second experiment examined whether antagonizing VIPR abolishes the effects of GLP-2 on lipid absorption during the fed state. As expected, in rats which received prior PBS (placebo control for VIPR antagonist), LFR increased from baseline and peaked at 1.5 h following GLP-2 treatment (PBS+GLP-2), while LFR remained relatively unchanged over time following PBS treatment (PBS+PBS) (Figure 2A). As a result, cumulative lymph volume over time was higher in rats treated with GLP-2 compared with placebo (*p* < 0.05, PBS+GLP-2 vs. PBS+PBS) (Figure 2B). In the groups treated with the VIPR antagonist (VIPA), GLP-2 also increased cumulative lymph volume compared with placebo (*p* < 0.05, VIPA+GLP-2 vs. VIPA+PBS). In the groups treated with GLP-2, LFR and cumulative lymph volume in rats treated with VIPA were not significantly different from those in rats treated with placebo (Figure 2A,B). TG output and ApoB output followed a similar pattern as LFR (Figure 2C,E) and cumulative TG amount was higher in rats pre-treated with PBS following GLP-2 compared with placebo (*p* < 0.05, PBS+GLP-2 vs. PBS+PBS). In the two GLP-2 treated groups (VIPA+GLP-2 vs. PBS+GLP-2), no statistical significance was detected between VIPA and placebo for TG output (Figure 2C), cumulative TG amount (Figure 2D), ApoB output (Figure 2E) and cumulative ApoB amount (Figure 2F). IL-22 concentration and output in lymph over time (Figure 2G,H) and IL-22 concentration in plasma at the end of the experiment (Figure 2I) were not significantly different among groups. The mRNA expressions of the genes for fatty acid transporters, lipid metabolism and IL-22 were not significantly different among groups (Figure 2J).

### 2.3. Antagonizing VIP Receptors Reduced GLP-2-Mediated Lymph Flow and Chylomicron Output During Post-Absorptive State

**Figure 3:**
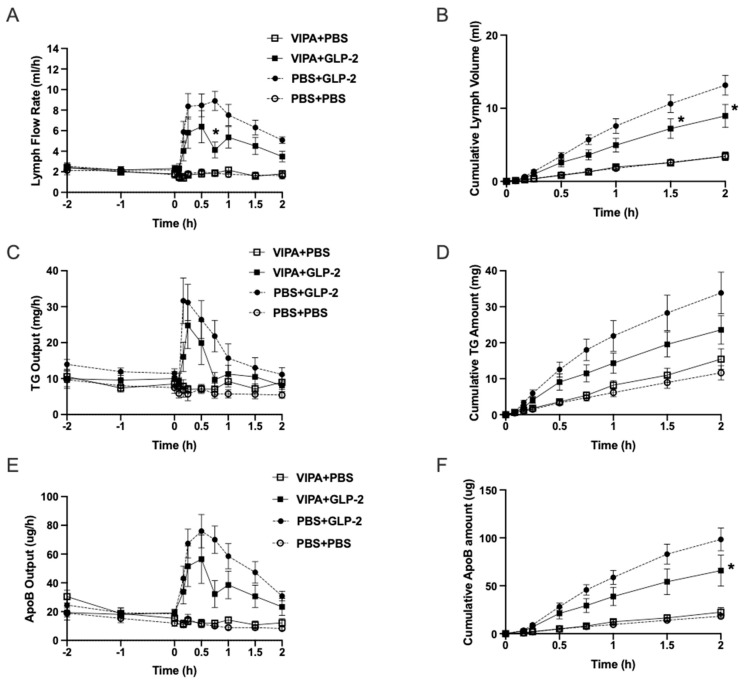
Effect of exogenous GLP−2 treatment on lipid release from the intestine in rats with VIP receptor antagonist during the post-absorptive state. (**A**) LFR over time. LFR was measured by collecting lymph for 2 h. (**B**) Cumulative lymph volume. (**C**) TG output and (**D**) cumulative TG amount in lymph were measured over 2 h. (**E**) ApoB output and (**F**) cumulative ApoB amount in lymph over time. Results are expressed as mean ± SEM: ip VIPA+PBS, n = 9; ip VIPA+GLP−2, n = 8; ip PBS+GLP−2, n = 9; ip PBS+PBS, n = 9. * *p* < 0.05, VIPA+GLP−2 vs. PBS+GLP−2. For clarity, only statistical significance for comparisons between VIPA and its control (PBS) in groups treated with GLP-2 is shown; statistical significance for increases from baseline within each treatment is not shown.

The third experiment examined the effects of VIPR antagonism on the mobilization of intestinal lipids by GLP-2 during the fasted state. LFR (Figure 3A), cumulative lymph volume (Figure 3B), TG output (Figure 3C), and cumulative TG amount (Figure 3D) were significantly higher in the GLP-2+PBS group compared to the PBS+PBS group. LFR in rats treated with VIPA+GLP-2 was significantly lower compared to PBS+GLP-2 at 45 min following GLP-2 administration (*p* < 0.05, VIPA+GLP-2 vs. PBS+GLP-2) (Figure 3A). Cumulative lymph volume was significantly lower in VIPA compared to its placebo control in GLP-2 groups (*p* < 0.05 VIPA+GLP-2 vs. PBS+GLP-2) (Figure 3B). TG output (Figure 3C) and cumulative TG amount were not significantly different between the two groups which received GLP-2 treatment (Figure 3D). In the two groups which received GLP-2, ApoB output (ng/h) was not significantly different between VIPA and its placebo control, despite a lower trend with VIPA vs. PBS (Figure 3E). Cumulative ApoB amount (ng) was significantly lower with VIPR antagonism than control at the end of experiment (*p* < 0.05 VIPA+GLP-2 vs. PBS+GLP-2) (Figure 3F).

## 3. Discussion

GLP-2 plays important roles in lipid handling in the intestine during both postprandial and post-absorptive states. During the postprandial state, it enhances lipid absorption. During the post-absorptive state, it promotes the release of lipids stored in the intestine 5–10 h after a meal intake. Several neural mechanisms have been proposed for lipid absorption and mobilization of stored lipids in response to exogenous GLP-2. Although previous studies support roles of the ENS, the key enteric neuronal components in these pathways remain undefined. In the present study in mesenteric lymph duct cannulated rats, we examined VIP signaling, an important component of the ENS, as a potential mediator of GLP-2’s effects on intestinal lipid handling. We found that peripheral VIP signaling is involved in GLP-2-mediated lipid mobilization but not absorption. Specifically, we showed that antagonizing VIPRs attenuated the effect of exogenous GLP-2 on releasing intestinally stored lipids. This finding expands the understanding of GLP-2’s mechanism of action in regulating intestinal lipid handling.

We first showed that exogenous VIP had minimal effects on lipid output during the postprandial state. This finding with pharmacological intervention contrasts with a study in genetic mouse models, where VIPR knockout reduced lipid absorption [17]. Reduced lipid absorption in VIPR knockout mice was accompanied by decreased expression of fatty acid transporter genes [17]. In our acute study in rats, no changes in mRNA levels of lipid transport and metabolism genes were detected. However, we detected an increase in IL-22 gene expression in the jejunum and IL-22 levels in lymph 2 h after VIP treatment. This is in line with another study showing that VIP increased IL-22 secretion by type-3 innate lymphoid cells isolated from mice [18]. Ours is the first study that measured IL-22 in lymph during lipid absorption following VIP treatment in vivo. Considering the time elapsed following VIP treatment, these effects may not be attributed to VIP; instead, they may be indirect responses to lipid absorption. The physiological significance of IL-22 in lymph warrants further study.

We then tested whether VIP signaling mediates GLP-2’s effects on lipid absorption by administering a VIPR antagonist. In the absence of the antagonist, GLP-2 stimulated lipid output compared to placebo treatment, in line with previous studies [1,2]. VIPR antagonism did not abrogate the enhancement of lipid output by GLP-2. In line with this, VIPR antagonism did not affect mRNA expression of key fatty acid transport or lipid metabolism genes in the jejunum. IL-22 mRNA in the jejunum and protein levels in both lymph and blood were also not affected. These results suggest that peripheral GLP-2’s actions on lipid absorption are independent of VIP and IL-22 signaling.

We further assessed whether VIP signaling mediates GLP-2’s effects on lipid mobilization by administering a VIPR antagonist. In the absence of the antagonist, GLP-2 resulted in enhanced lipid output compared to placebo, as in previous studies [3,5,19,20]. Pretreatment with the VIPR antagonist attenuated GLP-2-mediated increases in lipid output, supporting the involvement of VIP signaling in this process. Several possible mechanisms may underlie this effect. One possibility is through VIP signaling modulating lymphatic functions. GLP-2 mobilization of intestinal lipid stores involves modulation of lymphatic functions [10,21] and lymphatic functions are subject to the regulation by VIP signaling [22,23]. GLP-2R co-localizes with VIP-positive enteric neurons in the intestine [24,25] and chronic treatment with GLP-2 increases the number of VIP-expressing neurons [12,16,26]. Furthermore, GLP-2-induced gastric relaxation, gastric motility [27,28], and anti-inflammatory processes [16] are mediated by VIP released from enteric neurons. In our study, pretreatment with a VIPR antagonist attenuated the increase in lymph flow in response to GLP-2 during the post-absorptive state. It is therefore possible that GLP-2 stimulates lymphatic function in part through modulating VIP-expressing neurons. It is noted that regulation of lymphatic functions by VIP signaling may be context-dependent, as the VIPR antagonist alone did not elicit any effect on lymph flow during either postprandial or post-absorptive states. In line with this, the VIPR antagonist did not abolish a GLP-2-induced inhibitory effect on the motility of isolated murine proximal colon [24] or abrogate GLP-2-mediated increase in mesenteric blood flow in rats [29]. Another possible mechanism is that VIP signaling may mediate GLP-2 mobilization of intestinal lipid stores through the regulation of the ENS. GLP-2 mobilizes intestinal lipid stores in part by acting through the ENS [10]. Specifically, GLP-2 increased lipid secretion by stimulating the lymphatic contractility that was abolished with the ENS inhibitor mecamylamine [10]. As discussed above, VIP is secreted from enteric neurons and GLP-2 activates VIP-secreting neurons. Strong interactions exist between VIP and nitric oxide (NO) signaling in enteric neurons. In rats, VIP release from enteric neurons is regulated by NO [30]. Additionally, VIP and NO regulate jejunal net fluid absorption in rats with experimental colitis [31]. VIP is colocalized with NOS in inhibitory motor neurons [32,33] and GLP-2 can act via neuronal NOS to regulate lipid absorption during the fed state [9]. A link between GLP-2 and VIP or NOS has not been established before for intestinal lipid handling during the fasted state. Here our study shows that GLP-2 promotes the release of intestinally stored lipids in part through VIP signaling during the fasted state. Further investigations are needed to elucidate the exact mechanisms whereby VIP signaling regulates lymphatic functions and interacts with GLP-2 signaling during fed and fasted states.

The study has several limitations. First, it employed acute pharmacological interventions and short time-course sampling. Second, although the mesenteric lymph duct cannulated rats model allows for direct assessment of intestinal lipoprotein secretion, the findings should be interpreted with caution from this model alone. Future studies are warranted to evaluate the roles of VIP signaling in a chronic setting and with genetic models.

## 4. Materials and Methods

### 4.1. Peptides

[Gly^2^]GLP-2 (1–33) (a dipeptidyl peptidase IV-resistant GLP-2 analog, referred to as GLP-2) was obtained from Pepceuticals Ltd. (Leicestershire, UK). The VIP analog was obtained from Bachem (Hauptstrasse, Switzerland). The VIP antagonist was obtained from Bachem (Saint Helens, UK).

### 4.2. Animals

Adult male Sprague-Dawley rats (200–350 g body weight, 8–10 weeks old) were obtained from Charles River Laboratories (Senneville, QC, Canada). Rats were housed in pairs in polycarbonate cages in a room with controlled temperature, humidity and automatic 12 h light/dark cycle, with ad libitum access to water and standard laboratory diet (LabDiet, St. Louis, MO, USA; Prolab RMH 3000; calories provided by protein 26.1%, fat 14.4%, carbohydrates 59.5%). They were acclimatized in the above conditions for 2 weeks before surgery. All animal procedures were approved by the Animal Research Ethics Board of the University of Saskatchewan (Animal Use Protocol 20200080, 1/11/2021). All experimental methods and procedures involving animals adhered to the guidelines and regulations of the University Animal Care Committee of the University of Saskatchewan and complied with the ARRIVE guidelines.

### 4.3. Assessment of Lipid Absorption in Response to VIP

To assess the effects of VIP on lipid absorption, rats were pre-treated with exogenous VIP prior to providing lipids into the small intestine. Rats (Sprague-Dawley, 280–360 g, n = 8/group) were surgically implanted with id, ip and mesenteric lymph duct (MLD) catheters. Rats were randomly divided into two groups to receive a bolus of either VIP (ip, 10 nmol in 1 mL PBS) [16] or placebo (PBS). A total of 1 h later, they received an id fat load (Intralipid 20%, 1.5 mL, t = 0) into the duodenum. Lymph samples were collected through the MLD catheter at different time points (30′, 60′, 90′, 120′, 3 h and 4 h afterwards). At the end of the lymph collection, rats were euthanized by pentobarbital overdose. Blood samples were immediately collected via cardiac puncture and centrifuged at 2000× *g* for 10 min to isolate plasma. Jejunum tissues were also immediately collected upon euthanasia. Plasma and tissues were frozen at −80 °C until further analysis.

Lymph flow rates and TG concentration were measured. Cumulative lymph volume, TG output and cumulative TG amount were calculated as mentioned earlier. Lymph ApoB concentration (ng/mL) was measured using a rat ApoB ELISA kit (Cloud-Clone Corp., Katy, TX, USA). ApoB output and cumulative amount were calculated as mentioned earlier.

The concentrations of IL-22 (pg/mL) in the lymph and plasma were measured using a mouse/rat IL-22 Quantikine ELISA kit (Minneapolis, MN, USA). IL-22 output (pg/h) in lymph was calculated as the product of LFR and IL-22 concentration. Comparisons were made between VIP and control (placebo) groups.

Tissues were homogenized using the OMNI bead mill homogenizer. qPCR was performed on *Cd36*, *Fatp4*, *Fabp2*, *Apoa4*, *Mttp*, *Fos*, *Dgat1* and *Il22* mRNAs which were normalized to *Gapdh*.

### 4.4. Assessment of Lipid Absorption in Response to VIPR Antagonist

To assess the effects of VIP signaling on the regulation of lipid absorption by GLP-2, rats were pre-treated with exogenous VIPA prior to exogenous GLP-2 and lipid supply to the small intestine. Rats (Sprague-Dawley, 280–360 g, n = 7–9/group, 4 groups) were surgically implanted with id, ip and MLD catheters. Rats were first randomly assigned to 2 groups to receive either a placebo (ip PBS, 1 mL) or VIPR antagonist (ip, 85 ug in 1 mL PBS) [16]. A total of 1 h later, rats in each group were further randomized into 2 groups to receive either GLP-2 (ip, 75 ug, 1 mL) or a placebo (ip PBS, 1 mL). Additionally, all rats then received an id fat load (Intralipid 20%, 1.5 mL, t = 0). Lymph samples were collected through the MLD catheter at different time points, at 30′, 60′, 90′, 120′, 3 h and 4 h thereafter. Blood and tissue were collected as described above.

Lymph flow rates and TG concentration were measured. Cumulative lymph volume, TG output and Cumulative TG amount were calculated as earlier. ApoB concentration (ng/mL) was measured using a rat ApoB ELISA kit (Cloud-Clone Corp., Katy, TX, USA). ApoB output (ng/h) and cumulative ApoB amount were calculated as mentioned earlier.

The concentrations of IL-22 (pg/mL) in the lymph and plasma were measured using mouse/rat IL-22 Quantikine ELISA kit (Minneapolis, MN, USA). IL-22 output (pg/h) in lymph was calculated as the product of LFR and IL-22 concentration.

qPCR was performed on *Cd36*, *Fatp4*, *Fabp2*, *Apoa4*, *Mttp*, *Fos*, *Dgat1* and *Il22* mRNAs which were normalized to *Gapdh*.

### 4.5. Assessment of Lipid Mobilization in Response to VIPR Antagonist

To assess the effects of VIP signaling on the regulation of lipid mobilization by GLP-2, rats were provided with lipids into the small intestine. Lipid secretion was measured 5 h later, corresponding to the fasted state, following pretreatment with exogenous VIPA prior to exogenous GLP-2. Rats (Sprague-Dawley, 280–360 g, n = 8–9/group, 4 groups) were surgically implanted with i.d., i.p. and MLD catheters. All rats first received Intralipid infusion into the duodenum. Additionally, 4 h after, rats were randomized into 2 groups to receive a bolus of either the VIPR antagonist (i.p., 85 ug in 1 mL PBS) [16] or placebo (i.p. PBS, 1 mL). A total of 1 additional hour later, rats in each group were further randomized into 2 groups to receive a bolus of either GLP-2 (i.p., 75 ug, 1 mL) or a placebo (i.p. PBS, 1 mL). Lymph samples were collected before and 5, 10, 15, 30, 45, 60, 90 and 120 min after treatment with GLP-2 or PBS. Lymph flow rates and TG concentration were measured as above. Cumulative lymph volume, TG output and cumulative TG amount were calculated as above.

### 4.6. Statistics

All the data were presented as mean ± SEM. Data were analyzed and plotted using GraphPad Software (Version 9).

In the absorption study comparing VIP vs. placebo, time-course curves (including LFR, TG output, ApoB48 output, IL-22 concentration, and IL-22 output) were analyzed using two-way mixed ANOVA followed by Bonferroni’s multiple comparison test. Cumulative values (including lymph volume, TG amount, and ApoB48 amount) were analyzed using the unpaired *t*-test. IL-22 concentration in plasma was analyzed using the Mann–Whitney test. mRNA expression patterns were analyzed using two-way ANOVA followed by Bonferroni’s multiple comparison test. A *p* < 0.05 was considered statistical significance.

In the absorption study examining the effect of VIP receptor antagonism on effect of GLP-2, time-course curves (including LFR, TG output, ApoB48 output, IL-22 concentration, and IL-22 output) were analyzed using two-way mixed ANOVA followed by Sidak’s multiple comparison test. Cumulative values (including lymph volume, TG amount, and ApoB48 amount) were analyzed using one-way ANOVA followed by Bonferroni’s multiple comparison test. IL-22 concentration in plasma was analyzed using one-way ANOVA followed by Sidak’s multiple comparison test. mRNA expression patterns were analyzed using two-way ANOVA followed by Bonferroni’s multiple comparison test. A *p* < 0.05 was considered statistical significance.

In the mobilization study examining the effect of VIP receptor antagonism on the effect of GLP-2, time-course curves (including LFR, TG output, cumulative lymph volume, cumulative TG amount, ApoB48 output, and cumulative ApoB48 amount) were analyzed using two-way mixed ANOVA followed by Sidak’s multiple comparison test. A *p* < 0.05 was considered statistical significance.

## 5. Conclusions

Our results indicated that VIP signaling plays a role in GLP-2-mediated lipid mobilization during the post-absorptive state. This finding further expands our understanding of intestinal lipid storage and release dynamics and GLP-2 biology, which may help identify potential therapeutic approaches towards metabolic diseases and complications.

## Data Availability

No new data were created or analyzed in this study. Data sharing is not applicable to this article.

## Abbreviations

The following abbreviations are used in this manuscript:

ApoB	Apolipoprotein B
ENS	Enteric Nervous System
GLP-2	Glucagon-Like Peptide-2
GLP-2R	Glucagon-Like Peptide-2 Receptor
IL-22	Interleukin 22
LFR	Lymph Flow Rate
MLD	Mesenteric Lymph Duct
NO	Nitric Oxide
NOS	Nitric Oxide Synthase
TG	Triglyceride
VIP	Vasoactive Intestinal Peptide
VIPA	Vasoactive Intestinal Peptide Receptor Antagonist
VIPR	Vasoactive Intestinal Peptide Receptor

## References

[1] Hsieh J., Longuet C., Maida A., Bahrami J., Xu E., Baker C.L., Brubaker P.L., Drucker D.J., Adeli K. (2009). Glucagon-Like Peptide-2 Increases Intestinal Lipid Absorption and Chylomicron Production via CD36. Gastroenterology.

[2] Meier J.J., Nauck M.A., Pott A., Heinze K., Goetze O., Bulut K., Schmidt W.E., Gallwitz B., Holst J.J. (2006). Glucagon-Like Peptide 2 Stimulates Glucagon Secretion, Enhances Lipid Absorption, and Inhibits Gastric Acid Secretion in Humans. Gastroenterology.

[3] Dash S., Xiao C., Morgantini C., Connelly P.W., Patterson B.W., Lewis G.F. (2014). Glucagon-Like Peptide-2 Regulates Release of Chylomicrons from the Intestine. Gastroenterology.

[4] Hsieh J., Trajcevski K.E., Farr S.L., Baker C.L., Lake E.J., Taher J., Iqbal J., Hussain M.M., Adeli K. (2015). Glucagon-Like Peptide 2 (GLP-2) Stimulates Postprandial Chylomicron Production and Postabsorptive Release of Intestinal Triglyceride Storage Pools via Induction of Nitric Oxide Signaling in Male Hamsters and Mice. Endocrinology.

[5] Stahel P., Xiao C., Davis X., Tso P., Lewis G.F. (2019). Glucose and GLP-2 (Glucagon-Like Peptide-2) Mobilize Intestinal Triglyceride by Distinct Mechanisms. Arterioscler. Thromb. Vasc. Biol..

[6] Mukherjee K., Xiao C. (2024). GLP-2 Regulation of Intestinal Lipid Handling. Front. Physiol..

[7] Syed-Abdul M.M., Tian L., Xiao C., Lewis G.F. (2022). Lymphatics—Not Just a Chylomicron Conduit. Curr. Opin. Lipidol..

[8] Pedersen J., Pedersen N.B., Brix S.W., Grunddal K.V., Rosenkilde M.M., Hartmann B., Ørskov C., Poulsen S.S., Holst J.J. (2015). The Glucagon-like Peptide 2 Receptor Is Expressed in Enteric Neurons and Not in the Epithelium of the Intestine. Peptides.

[9] Grande E.M., Raka F., Hoffman S., Adeli K. (2022). GLP-2 Regulation of Dietary Fat Absorption and Intestinal Chylomicron Production via Neuronal Nitric Oxide Synthase (NNOS) Signaling. Diabetes.

[10] Syed-Abdul M.M., Tian L., Samuel T., Wong A., Hong Y.-K., Dacosta R.S., Lewis G.F. (2024). Glucagon-Like-Peptide-2 Stimulates Lacteal Contractility and Enhances Chylomicron Transport in the Presence of an Intact Enteric Nervous System. Gastro Hep Adv..

[11] Said S.I., Mutt V. (1970). Polypeptide with Broad Biological Activity: Isolation from Small Intestine. Science.

[12] de Heuvel E., Wallace L., Sharkey K.A., Sigalet D.L. (2012). Glucagon-like Peptide 2 Induces Vasoactive Intestinal Polypeptide Expression in Enteric Neurons via Phophatidylinositol 3-Kinase-γ Signaling. Am. J. Physiol.-Endocrinol. Metab..

[13] Dharmsathaphorn K., Harms V., Yamashiro D.J., Hughes R.J., Binder H.J., Wright E.M. (1983). Preferential Binding of Vasoactive Intestinal Polypeptide to Basolateral Membrane of Rat and Rabbit Enterocytes. J. Clin. Investig..

[14] Spessert R. (1993). Vasoactive Intestinal Peptide Stimulation of Cyclic Guanosine Monophosphate Formation: Further Evidence for a Role of Nitric Oxide Synthase and Cytosolic Guanylate Cyclase in Rat Pinealocytes. Endocrinology.

[15] González C., Barroso C., Martín C., Gulbenkian S., Estrada C. (1997). Neuronal Nitric Oxide Synthase Activation by Vasoactive Intestinal Peptide in Bovine Cerebral Arteries. J. Cereb. Blood Flow Metab..

[16] Sigalet D.L., Wallace L.E., Holst J.J., Martin G.R., Kaji T., Tanaka H., Sharkey K.A. (2007). Enteric Neural Pathways Mediate the Anti-Inflammatory Actions of Glucagon-like Peptide 2. Am. J. Physiol.-Gastrointest. Liver Physiol..

[17] Talbot J., Hahn P., Kroehling L., Nguyen H., Li D., Littman D.R. (2020). Feeding-Dependent VIP Neuron–ILC3 Circuit Regulates the Intestinal Barrier. Nature.

[18] Seillet C., Luong K., Tellier J., Jacquelot N., Shen R.D., Hickey P., Wimmer V.C., Whitehead L., Rogers K., Smyth G.K. (2020). Author Correction: The Neuropeptide VIP Confers Anticipatory Mucosal Immunity by Regulating ILC3 Activity. Nat. Immunol..

[19] Mukherjee K., Wang R., Xiao C. (2024). Release of Lipids Stored in the Intestine by Glucagon-Like Peptide-2 Involves a Gut-Brain Neural Pathway. Arterioscler. Thromb. Vasc. Biol..

[20] Mukherjee K., Khan M.S.A., Howland J.G., Xiao C. (2025). Glucagon-like Peptide-2 Acts Partially Through Central GLP-2R and MC4R in Mobilizing Stored Lipids from the Intestine. Nutrients.

[21] Syed-Abdul M.M., Stahel P., Tian L., Xiao C., Nahmias A., Lewis G.F. (2022). Glucagon-like Peptide-2 Mobilization of Intestinal Lipid Does Not Require Canonical Enterocyte Chylomicron Synthetic Machinery. Biochim. Biophys. Acta (BBA)-Mol. Cell Biol. Lipids.

[22] von der Weid P., Rehal S., Dyrda P., Lee S., Mathias R., Rahman M., Roizes S., Imtiaz M.S. (2012). Mechanisms of VIP-induced Inhibition of the Lymphatic Vessel Pump. J. Physiol..

[23] Bojö L., Lefebvre R.A., Nellgård P., Cassuto J. (1993). Involvement of Vasoactive Intestinal Polypeptide in Gastric Reflex Relaxation. Eur. J. Pharmacol..

[24] Amato A., Rotondo A., Cinci L., Baldassano S., Vannucchi M.G., Mulè F. (2010). Role of Cholinergic Neurons in the Motor Effects of Glucagon-like Peptide-2 in Mouse Colon. Am. J. Physiol.-Gastrointest. Liver Physiol..

[25] Cinci L., Faussone-Pellegrini M.S., Rotondo A., Mulè F., Vannucchi M.G. (2011). GLP-2 Receptor Expression in Excitatory and Inhibitory Enteric Neurons and Its Role in Mouse Duodenum Contractility. Neurogastroenterol. Motil..

[26] Sigalet D.L., Wallace L., De Heuval E., Sharkey K.A. (2010). The Effects of Glucagon-like Peptide 2 on Enteric Neurons in Intestinal Inflammation. Neurogastroenterol. Motil..

[27] Amato A., Baldassano S., Serio R., Mulè F. (2009). Glucagon-like Peptide-2 Relaxes Mouse Stomach through Vasoactive Intestinal Peptide Release. Am. J. Physiol.-Gastrointest. Liver Physiol..

[28] Traini C., Idrizaj E., Garella R., Squecco R., Vannucchi M.G., Baccari M.C. (2020). Glucagon-like Peptide-2 Interferes with the Neurally-Induced Relaxant Responses in the Mouse Gastric Strips through VIP Release. Neuropeptides.

[29] Galsgaard K.D., Hartmann B., Rosenkilde M.M., Holst J.J., Gasbjerg L.S., Sørensen C.M. (2025). GLP-2 and GIP Acutely Increase Superior Mesenteric Artery Blood Flow in Male Rats, and the Effect Is Independent of Nitric Oxide and Vasoactive Intestinal Peptide. Physiol. Rep..

[30] Allescher H.D., Kurjak M., Huber A., Trudrung P., Schusdziarra V. (1996). Regulation of VIP Release from Rat Enteric Nerve Terminals: Evidence for a Stimulatory Effect of NO. Am. J. Physiol.-Gastrointest. Liver Physiol..

[31] Mourad F.H., Barada K.A., Bou Rached N.A., Khoury C.I., Saadé N.E., Nassar C.F. (2006). Inhibitory Effect of Experimental Colitis on Fluid Absorption in Rat Jejunum: Role of the Enteric Nervous System, VIP, and Nitric Oxide. Am. J. Physiol.-Gastrointest. Liver Physiol..

[32] Furness J.B., Pompolo S., Shuttleworth C.W.R., Burleigh D.E. (1992). Light- and Electron-Microscopic Immunochemical Analysis of Nerve Fibre Types Innervating the Taenia of the Guinea-Pig Caecum. Cell Tissue Res..

[33] Keef K.D., Shuttleworth C.W.R., Xue C., Bayguinov O., Publicover N.G., Sanders K.M. (1994). Relationship between Nitric Oxide and Vasoactive Intestinal Polypeptide in Enteric Inhibitory Neurotransmission. Neuropharmacology.

